# Impact of 5HydroxyMethylCytosine (5hmC) on reverse/direct association of cell-cycle, apoptosis, and extracellular matrix pathways in gastrointestinal cancers

**DOI:** 10.1186/s12863-022-01061-x

**Published:** 2022-06-29

**Authors:** Sayyed Sajjad Moravveji, Samane Khoshbakht, Majid Mokhtari, Mahdieh Salimi, Hossein Lanjanian, Sajjad Nematzadeh, Mahsa Torkamanian-Afshar, Ali Masoudi-Nejad

**Affiliations:** 1 Laboratory of Systems Biology and Bioinformatics (LBB), Department of Bioinformatics, Kish International Campus, University of Tehran, Kish Island, Iran; 2 Department of Medical Genetics, Institute of Medical Biotechnology, National Institute of Genetic Engineering and Biotechnology (NIGEB), Tehran, Iran; 3 Molecular Biology and Genetics Department, Engineering and Natural Science Faculty, Istinye University, Istanbul, Turkey; 4 Computer Engineering Department, Architecture and Engineering Faculty, Nisantasi University, Istanbul, Turkey; 5 Laboratory of Systems Biology and Bioinformatics (LBB), Institute of Biochemistry and Biophysics, University of Tehran, Tehran, Iran

**Keywords:** cfDNA, 5-hydroxymethylcytosine, Network, Predictive model, Gastrointestinal cancers

## Abstract

**Background:**

Aberrant levels of 5-hydroxymethylcytosine (5-hmC) can lead to cancer progression. Identification of 5-hmC-related biological pathways in cancer studies can produce better understanding of gastrointestinal (GI) cancers.

We conducted a network-based analysis on 5-hmC levels extracted from circulating free DNAs (cfDNA) in GI cancers including colon, gastric, and pancreatic cancers, and from healthy donors. The co-5-hmC network was reconstructed using the weighted-gene co-expression network method. The cancer-related modules/subnetworks were detected. Preservation of three detected 5-hmC-related modules was assessed in an external dataset. The 5-hmC-related modules were functionally enriched, and biological pathways were identified. The relationship between modules was assessed using the Pearson correlation coefficient (*p*-value < 0.05). An elastic network classifier was used to assess the potential of the 5-hmC modules in distinguishing cancer patients from healthy individuals. To assess the efficiency of the model, the Area Under the Curve (AUC) was computed using five-fold cross-validation in an external dataset.

**Results:**

The main biological pathways were the cell cycle, apoptosis, and extracellular matrix (ECM) organization. Direct association between the cell cycle and apoptosis, inverse association between apoptosis and ECM organization, and inverse association between the cell cycle and ECM organization were detected for the 5-hmC modules in GI cancers. An AUC of 92% (0.73–1.00) was observed for the predictive model including 11 genes.

**Conclusion:**

The intricate association between biological pathways of identified modules may reveal the hidden significance of 5-hmC in GI cancers. The identified predictive model and new biomarkers may be beneficial in cancer detection and precision medicine using liquid biopsy in the early stages.

**Supplementary Information:**

The online version contains supplementary material available at 10.1186/s12863-022-01061-x.

## Background

Circulating free DNAs (cfDNA) are free DNA fragments found in plasma, serum, or other body fluids. Physiological events such as apoptosis or micrometastasis are the origin of cfDNAs in the body [1]. Previous studies have shown that cfDNA levels are 2–3 times greater in cancer patients than in healthy individuals [2]. Use of cfDNA in cancer monitoring has emerged with the advent of liquid biopsy [3]. In cancer studies, cfDNAs are investigated for genomics and epigenomics patterns as they are representative of the primary tumor, and because their levels can be assessed noninvasively. Thus, they may be a feasible alternative to biomarker detection in the clinic  [1]. In cfDNA studies, 5-hydroxymethylcytosine (5-hmC) is a specific DNA modification used in cancer monitoring. However, it has rarely been applied in pan-cancer studies [4, 5]. Thus, we investigated 5-hmC-related gene clusters in gastrointestinal (GI) cancers.

5-hmC is an epigenetic mark originating from 5-methylcytosine, a cytosine modification found in DNA [6]. 5-hmC plays a significant role in biological processes such as gene expression and cancer pathogenesis [7]. The presence of cytosine modifications, such as 5-hmC in gene bodies is related to transcription level; however, the mechanism is unknown [7]. To the extent that cfDNAs indicate primary tumor characteristics, detecting clustered genes related to increases/decreases in 5-hmC may allow noninvasive determination of the mechanism of such cytosine modifications.

Biomarkers are an important topic in modern precision medicine, especially in cancer study [8]. 5-hmC is widely studied in cfDNAs as a promising cancer biomarker as it can be detected rapidly and noninvasively. Such markers in cancer research could lead to biomarkers or predictive models that can noninvasively classify normal and disease samples, which could be an integral part of cancer management in some cases [9–11].

In the context of systems biology, techniques and algorithms have been developed to study complex biological environments [12–14] and identify new and effective targets in the design of new drugs [11, 15, 16] or repurpose existing drugs for different treatments [17]. There are several methods for determining the biological mechanisms of 5-hmC-enriched genes. Network-based methods are promising for identifying clustered 5-hmC-enriched genes that activate specific biological pathways. Detecting such pathways could result in more precise treatments and deeper insight into cancer biology. Predictive models for cancer detection play an essential part in research. Assessing 5-hmC markers in cfDNAs may be of great interest to scientists and physicians in terms of biological insights and practical predictive models.

In this study, we aimed to identify co-5hydroxymethylcytosine (co-5hmC) modules, assess the biological relationships between them in GI cancers, and develop a diagnostic model that can distinguish between cancer patients and healthy individuals. First, differentially hydroxymethylated genes were detected, and the co-5hmC network was reconstructed using the weighted-gene co-expression network analysis (WGCNA) method. The co-5hmC network was clustered; the GI-related 5-hmC modules were detected, and the relation between co-5hmC modules was assessed. The gene significance (GS) values were used as the cut-off index to select the most cancer-related genes. To develop a diagnostic model based on 5-hmC values, the elastic network method was implemented and validated in an external dataset.

## Results

We detected hydroxymethylated modules to predict GI cancers, and investigated the biological patterns. We preprocessed the data using the WGCNA method to reconstruct a co-hydroxymethylated network, and extracted the hydroxymethylated modules related to GI cancers.

**Fig. 1:**
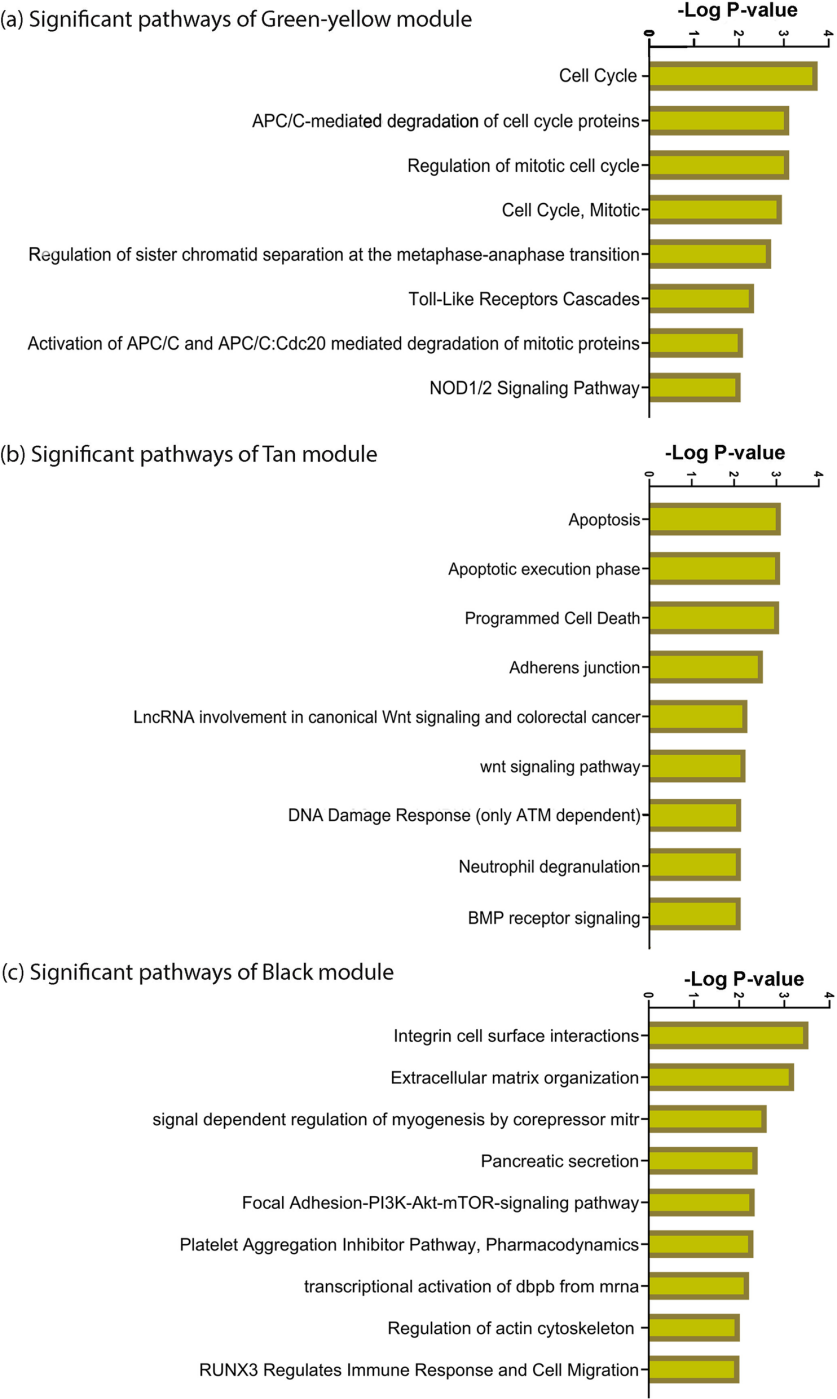
Biological pathways related to three statistically significant gastrointestinal cancer subnetworks/modules

Coefficient variation and differentially hydroxymethylated genes (DHMG) filters were used on the data; 7000 genes and all samples were included for downstream analyses. The network was reconstructed, and 14 modules were detected. Three modules had statistically trait-significant *p*-values (*p*-value < 0.05), with an absolute value of Pearson correlation greater than 0.5. The significant modules were denoted as GreenYellow, Tan, and Black, and included 94 genes, 91 genes, and 159 genes, respectively (Tables S1, S2, S3). These modules were functionally enriched; the most significant pathways are shown in Fig. 1 (*p*-value < 0.01). The GreenYellow module indicated biological pathways including ‘*Cell cycle’*, ‘*Toll-Like receptor cascades*’, and ‘*NOD1/2 signaling pathway*’ (Fig. 1a). The Tan module indicated biological pathways including ‘*Apoptosis’*, ‘*Adherens junction*’, ‘*DNA damage response (only ATM-dependent)*’, ‘*Neutrophil degranulation*’, and ‘*BMP receptor signaling*’ (Fig. 1b). The Black module indicated biological pathways including ‘*RUNX3 regulates immune response and cell migration*’, ‘*Extracellular matrix organization*’, ‘*Integrin cell surface interactions*’, and ‘*Transcriptional activation of dbpb from mRNA* (Fig. 1c).

### Module reproducibility

To demonstrate module preservation or reproducibility, we extracted two preservation statistics, ${Z}_{summary}$ and ${Median}_{rank}$. Both indicated that the hydroxymethylated GI-related modules were preserved. The preservation results for 25%, 50%, and 75% of the data (GSE81314), and GSE89570 are provided in Supplementary tables S5 and S6. The ${Z}_{summary}$ values for the GreenYellow module were 12, 15, 16, and 18 (${Z}_{summary}$ > 12 indicates highly preserved). The ${Z}_{summary}$ values for the Tan module were 12, 13, 15, and 16. The ${Z}_{summary}$ values for the Black module were 37, 35, 42, and 40. As the Black module was much larger than the GreenYellow and Tan modules, we extracted the ${Median}_{rank}$ statistics, which are less sensitive to module size. A smaller larg ${Median}_{rank}$ value indicates greater preservation. The ${Median}_{rank}$ values for the Black module were 4, 3, 8, and 3 out of 15. The ${Median}_{rank}$ values for the GreenYellow module were 7, 7, 6, and 6 out of 15. The ${Median}_{rank}$ values for the Tan module were 5, 3, 5, and 5 out of 15.

### Module association

**Fig. 2:**
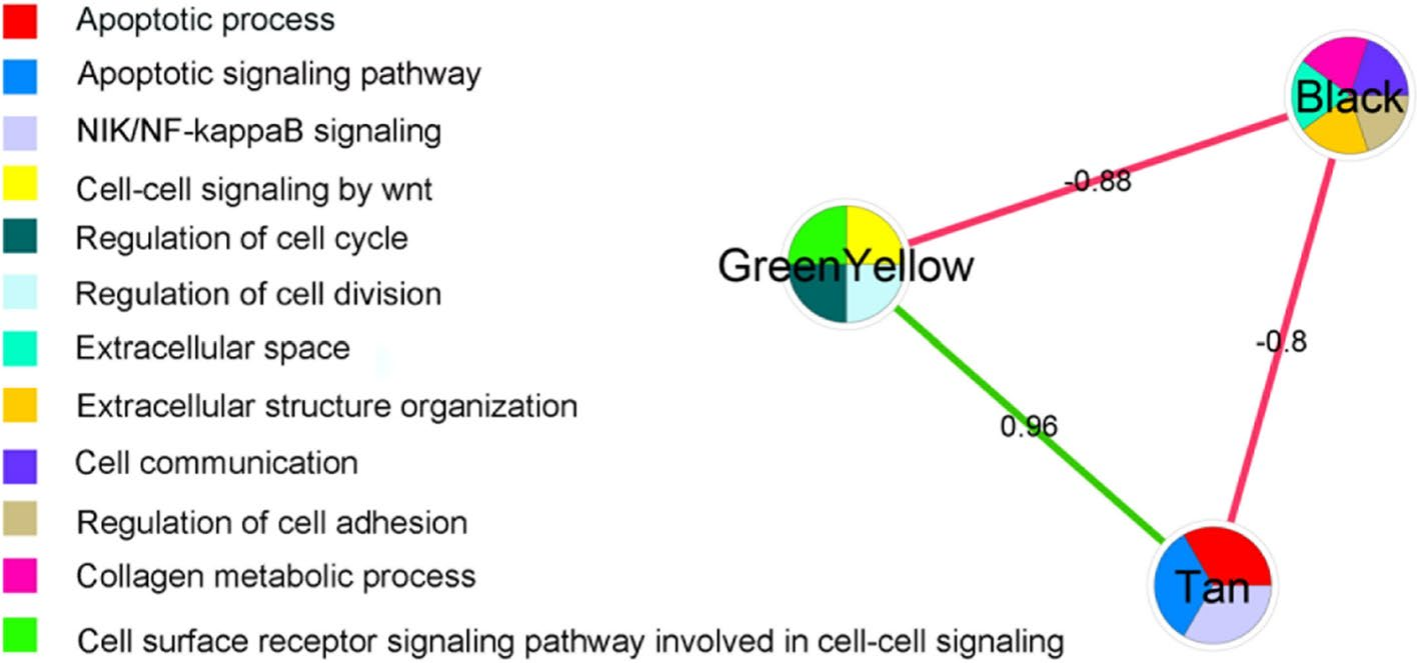
Inverse/direct interplay between 5-hmC gastrointestinal cancer subnetworks/modules. The node color represents the biological process. The numbers on the edges represent the PCC

The associations between GI-related modules were extracted using PCC. The association values and their biological processes are illustrated in Fig. 2. We detected a reverse association between the GreenYellow and Black modules (PCC = -0.88), a reverse association between the Black and Tan modules (Pearson Correlaion Coefficient (PCC) = -0.8), and a direct association between the GreenYellow and Tan modules (PCC = 0.96). We detected mostly cell cycle-related biological processes such as *regulation of cell cycle* and *regulation of cell division* for the GreenYellow module, apoptosis-related biological processes for the Tan module such as *apoptotic process*, and cell adhesion and extracellular-related biological processes for the Black module (Fig. 2).

### Predictive modules for detection of GI cancers in cfDNA

**Fig. 3:**
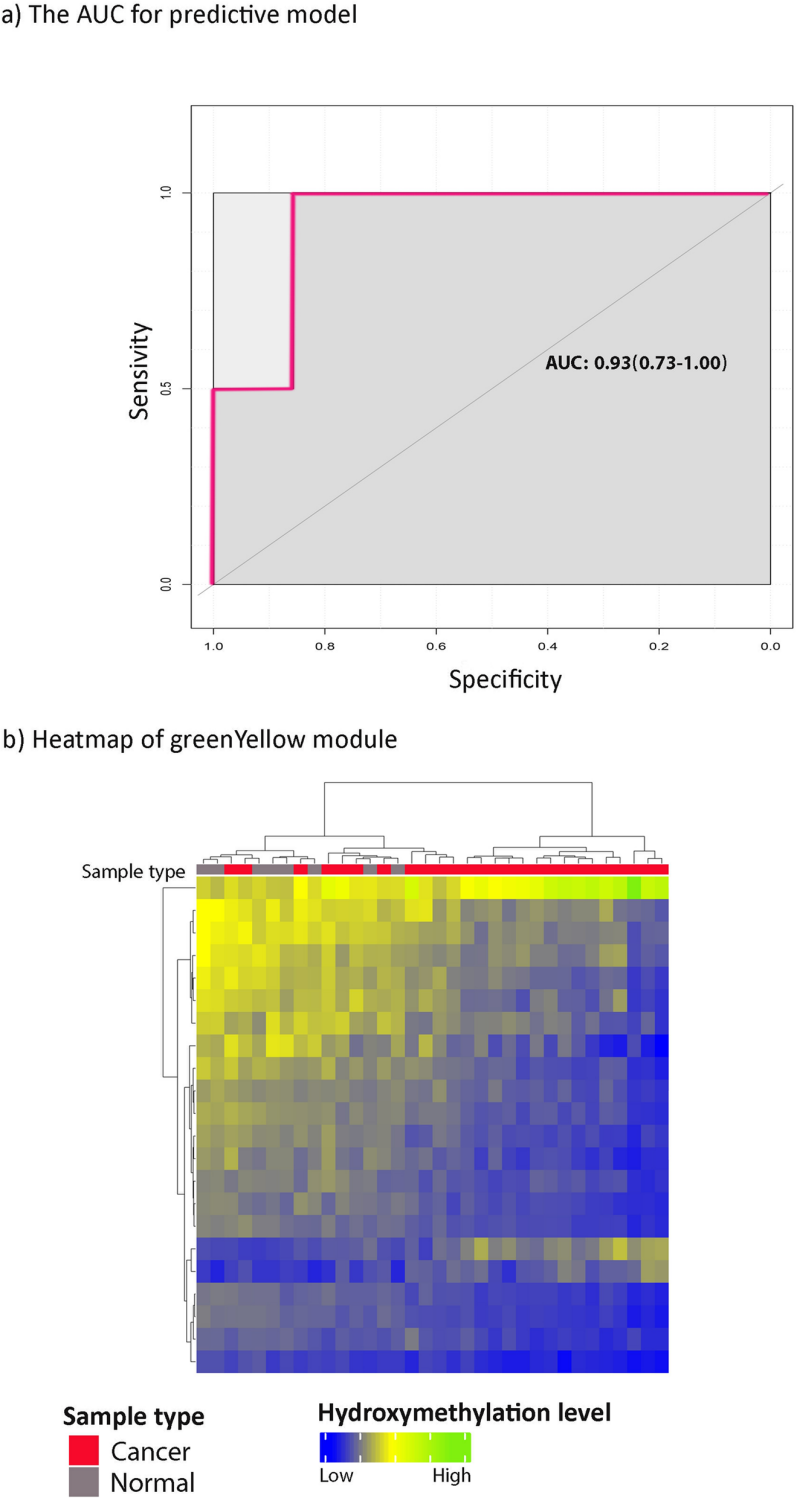
Distinguishing ability of 5-hmC gastrointestinal cancer modules/ subnetworks. **a **Area under the curve (AUC) for GreenYellow subnetwork/module. This Elastic Net model includes c21orf91, C4orf33, dbf4, gapt, gucy1b3, kiaa1468, klrg1, mthfd2l, samd11, sod3, and vrk1. **b **Heatmap of GreenYellow subnetwork/module. The heatmap includes 22 genes of the GreenYellow module with gene significance greater than 0.5, and 68 samples

To develop a predictive model for classifying cancer and normal cfDNA, we used the elastic net method for the GreenYellow, Tan, and Black hydroxymethylated modules. To reduce the feature space, we computed the gene significance (GS) for each gene; genes with a GS greater than 0.5 were selected as the input for the elastic net model (Table S1). In the GreenYellow module, 11 genes were selected as significant features for the elastic net. The model was trained, and the best alpha and lambda values were estimated as 0.216 and 0.048, respectively. The Area Under the Curve (AUC) was estimated as 92% (confidence interval: 0.731–1.000) (Fig. 3a). The final features extracted from the GreenYellow module (11 features) and the estimated coefficients are presented in Table S4. The final selected genes were *c21orf91*, *C4orf33*, *dbf4*, *gapt*, *gucy1b3*, *kiaa1468*, *klrg1*, *mthfd2l*, *samd11*, *sod3*, and *vrk1*. The gene expression changes of the final 11 genes were assessed in different GI cancers (Table 1) (FDR < 0.05); five of the 11 genes exhibited a significant change in gene expression in different GI cancers (Table 1).

**Table 1:** Gene expression changes

Gene stable ID	Gene name	Gene type	Gastric cancer	Colon cancer	Hepatocellular carcinoma
ENSG00000154642	C21orf91	protein_coding			-0.90
ENSG00000151470	C4orf33	protein_coding	2.04		
ENSG00000100749	VRK1	protein_coding	1.08	1.02	1.66
ENSG00000006634	DBF4	protein_coding		1.45	1.74
ENSG00000175857	GAPT	protein_coding		-1.47	

The numbers in the table indicate the logarithm of fold change (FDR < 0.05)

The other modules did not indicate a high AUC; thus, the GreenYellow module was selected for the final model. The clustering potential of the GreenYellow module was assessed using a heatmap; the GreenYellow module was able to cluster normal/cancer samples well (Fig. 3b). The heatmap includes 22 genes in the GreenYellow module with a GS greater than 0.5, and 68 samples (Table S1).

## Discussion

Although 5-Hydroxymethylcytosine signatures in cell-free DNAs provide information about tumors, the co-5-Hydroxymethylcytosine subnetworks/modules, their biological pathways, and the associations of biological processes in cfDNAs related to 5-hmC were not thoroughly investigated. 5-hydroxymethylated modules may be a cancer signature, and may be useful as clinical diagnostic biomarkers with liquid biopsy for a broad range of cancers [18, 19].

We reconstructed the 5-hydroxymethylated network and clustered it to detect the GI-cancer-related modules. The reproducibility of GI cancer-related modules was assessed [8, 11]. The biological pathways of significant modules were detected and visualized. To develop a predictive model for distinguishing GI patients from healthy individuals, the GS method and elastic net classifier were used for feature selection and model specification, respectively. The cross-validation, heatmap, and AUC were used for model validation.

cfDNAs are detectable using liquid biopsy, and could be used in the clinic to provide more precise therapies in the early stages [2, 20, 21]. Stratifying GI patients and healthy individuals using a limited number of genes would be useful and cost-effective, especially as a noninvasive method. In this study, the GreenYellow module was found to be GI-related; its potential in stratifying patients and healthy individuals was computationally validated using the elastic net model and hierarchical clustering (Fig. 3a, b). After experimental validation, it could be used in the clinic for cancer prediction.

The biological pathways and processes of three GI cancer-related modules, and their associations, were assessed in this study (Figs. 1, 2). The associations between cell-cycle arrest and apoptosis were independently investigated in several cancer-related studies [22–24]. In our study, a direct association between these metastasis-prone biological processes was detected for aberrant 5-hmC genes in GI cancers (Fig. 2). Close direct association may indicate concurrent activation of cell-cycle and apoptosis processes through 5-hmC aberrations in GI cancers.

In contrast, there is a close inverse association between cell-cycle-related biological processes and extracellular structure organization/cell adhesion-related biological processes (Fig. 2). In studies conducted by Walker et al. and Pickup et al., several aspects of extracellular matrix (ECM) dysregulation leading to cancer progression and metastasis were individually explored [25, 26]. It was reported that the ECM concentration increases and the ECM stiffens during tumor development. The stiffened ECM increases cell mobility and reduces the expression of genes that typically inhibit cell-cycle progression and proliferation [25, 26]. In our study, we detected an inverse association between ECM dysregulation and cell-cycle activity for the Black and GreenYellow hydroxymethylated modules (Fig. 2), which may be consistent with results reported by Walker et al. and Pickup et al. [25, 26]. However, they did not study cfDNAs. The *LUX* gene family secreted by primary tumor cells is responsible for ECM stiffness and total ECM concentration [25]. Some members of the *LUX* family were detected in our study, including *lux*, *lux1*, and *lux3*. They exhibited a low level of 5-hmC. An inverse association between the high volume of ECM stiffness and reduced cell cycle inhibition may be due to aberrant 5-hmC in primary cancer cells detected in cfDNAs.

Many previous studies have investigated the role of 5-hmC in the regulation of gene expression [4, 27–29]. Due to a lack of gene expression values, we were unable to assess the correlation between dysregulation of gene expression and aberrant 5-hmC values for detected genes in significant modules in this study. However, we assessed the gene expression changes of 11 final genes using TCGA data. Genes such as *samd11* and *mthfd2l* were reported as diagnostic 5-hmC signatures in previous studies [2, 30]. These biomarkers were detected in the GreenYellow and Black modules. Although there is no experimental evaluation in our study, on the basis of previous studies, the new genes detected in these modules may be new 5-hmC-related biomarkers for GI cancers.

The other significant finding in this study is a close inverse association between the Black and Tan modules, which indicate apoptosis and ECM organization and pathways, respectively. Signaling properties of the ECM such as three-dimensional organization of cells and ECM structures have significant impacts on cellular processes such as proliferation and apoptosis [31]. Moreover, the DNA damage response pathway, which is significant in the Tan module, is the vital determinant of the plasticity of the cellular genome and depends on signaling pathways that regulate apoptosis. Cell adhesion to the ECM, as a part of ECM organization, regulates several of these pathways [31, 32]. Less cell–ECM contact leads to more apoptosis. This finding is consistent with the results from two 5-hmC-related modules (Figs. 1, 2). We concluded that activation of ECM and apoptosis pathways may be related to aberrant 5-hmC values. Our findings for 5-hmC pathways were not experimentally validated; however, we computationally validated the reducibility in another dataset. Accordingly, some of the associations found in our study were reported in previous studies on 5-hmC in GI cancers, including the inverse associations between cell-cycle and ECM organization, and between apoptosis and ECM organization [27, 28]. However, some of the associations in GI cancers have not been reported, and are good targets for future research. Although we assessed the associations between biological pathways of GI-related modules, the module activities were not assessed in each GI-related cancer. Cubuk et al. assessed the module activities by integrating gene expression into biological pathways [33].

As cfDNAs mirror the primary characteristics of every patient, they may be an option for precision medicine in the clinic [21, 34]. In the future, checking the 5-hmC profile may be practical in precision medicine for GI cancers with aberrations, such as a patient’s specific 5-hmC. With 5-hmC-related modules and their associations, predictive models based on 5-hmC may be highly practical for cfDNAs as they are detectable using a noninvasive method. Such predictive models could be used in the clinic to distinguish patients from healthy individuals.

The studies by Song et al. and Li et al. [4, 18] were performed to detect cancer biomarkers using statistical methods including the fold change and t-test. In this study, we used a network-based method to detect GI-related 5-hmC modules, their associations, and 5-hmC biomarkers for GI cancers.

## Conclusions

The effect of 5-hmC in GI cancers is not fully understood. 5-hmC-related modules in gastrointestinal cancers, their biological pathways, and the associations among them might be used efficiently in the clinic, and also such studies can provide biological insights into GI cancers. 

## Methods

### Data and preprocessing

The GSE81314 and GSE89570 datasets were downloaded from the National Center for Biotechnology Information (NCBI). The 5-hydroxymethylcytosine (5-hmC) data were used for downstream analyses. GSE81314 includes 5-hmC methylation values for healthy donors (*n* = 16), lung cancer, hepatocellular carcinoma (*n *= 24), pancreatic cancer (*n* = 15), breast cancer (*n* = 6), colon cancer (*n* = 8), glioblastoma (*n* = 8), and gastric cancer (*n* = 9); breast, lung, and glioblastoma cancers were filtered out. In addition, we included 5-hmC profiles for 90 healthy individuals and 191 patients diagnosed with colorectal (*n* = 71), gastric (*n* = 61), pancreatic (*n* = 25), and liver (*n* = 34) cancer in our study from GSE89570 for computational validation.

The processed 5-hmC values were downloaded. For preprocessing, genes without values (NA) were filtered out. The coefficient of variation was computed for each gene; the first quartile of genes that had minimum variation among the samples was filtered out. After preprocessing, the FPKM values of 5-hmC were computed and median-centered using R [18]; the median-centered data are robust to outliers. Differentially hydroxymethylated genes (DHMGs) were detected at the probe level using the LIMMA package in R (FDR < 0.05, Benjamini Hochberg adjustment) [35]. The significant methylated genes were computed separately for each cancer type, and between healthy individuals and cancer patients. The significant DHMGs were combined to reconstruct the co-5hmC network.

To assess the gene expression changes of the final gene list, the gene expression of GI cancers was downloaded from the TCGA database [36] and normalized using edgeR package [37]. The differentially expressed genes were extracted [37].

### Network reconstruction and GS detection

We detected 5-hmC GI-related modules/subnetworks in cfDNAs. After preprocessing and detecting DMGs, the co-hydroxymethylated network was reconstructed using the WGCNA approach with the Pearson correlation coefficient (WGCNA version 1.71) [38]. As our data were spread around zero and the values were small, we did not use the scale-free part of the WGCNA approach (soft thresholding power = 1). The topological overlap matrix and connectivity measures were computed to detect more dense clusters [11, 38, 39] then, hierarchical clustering was used to detect modules. The minimum module size and deepSplit parameter were set as 30 and 4, respectively. For GI-related modules, the first principle of each module was computed as module eigengenes, and the Pearson correlation coefficients (PCC) between module eigengenes and cancer trait (cancer = 1/healthy = 0) were identified. Modules with a PCC greater than 0.5 and significant *p*-values were selected as GI-related modules.

To detect biological pathways and biological processes related to GI-cancer modules, we conducted overrepresentation analyses using the ConsensesPthDB webserver (*p*-value cutoff = 0.01, minimum overlap = 2). 

### Reproducibility of modules

Module reproducibility is important, and was checked with preservation statistics. To investigate the reproducibility of detected modules, we used the ${Z}_{summary}$ and ${Median}_{rank}$ statistics [11, 39]. As the validation datasets for preservation analysis were limited, we computed the preservation statistics for the external dataset GSE89570. We randomly selected 75%, 50%, and 25% of the samples from GSE81314 for the pattern preservation analyses.

${Z}_{summary}$ and ${Median}_{rank}$ were computed. A larger ${Z}_{summary}$ and smaller ${Median}_{rank}$ indicate greater module pattern preservation. A ${Z}_{summary}$ greater than 10 indicates preserved module patterns; there is no cutoff for ${Median}_{rank}$ [39]

### Association between modules

The association between modules was computed using the PCC for the first principle of each module. The first principle of a module is the representative containing the most information about the module/subnetwork.

### Predictive model for detection of GI cancers in cfDNA

As early detection of GI cancer is of great importance in the clinic, we developed a predictive model for classifying patients. We defined two labels for classification, cancer and normal. We included the significant modules GreenYellow, Tan, and Black as a feature space for three classification models. As there were sample size limitations compared to the feature space for all models, we used the elastic net classification model. The elastic net model is a regularized model that combines the least absolute shrinkage, selection operator (lasso), and ridge methods. There are two parameters in the elastic net, alpha and lambda. Lambda indicates the penalty effect, and alpha indicates the effects of norm 1 or norm 2 on the elastic model. If the value of alpha is 1, the lasso model is used. If the value of alpha is 0, the ridge model is used. To obtain the best alpha and lambda values, we used the cv.glmnet and cva.glmnet functions in R, which are cross-validation-based functions that find the optimum lambda and alpha values. Regularized models such as the elastic net are suitable for reducing overfitting. The area under the curve (AUC) for the models was computed for each module; the model with the greatest AUC was selected as the final model. In cross-validation, 75% of the samples were used to train the predictive model. The AUC was computed for the external dataset GSE89570. The GI-related cancer samples and healthy samples were included in the validation analysis (glmnet version 4.1.4, caret version 6.0.9, e1071 version 1.7.9, R version 4.0.5 64-bit).

Before using the elastic net on the modules, we computed the GS for three significant modules to indicate the correlation between the eigengene vector of the module and the trait (cancer/normal hydroxyl methylation). A greater GS indicates greater relation to the trait. We used the GS as a feature selection method for our classification model (cutoff = 0.5).

## Supplementary Information

**Additional file 1. **

## Data Availability

GSE81314, GSE89570. https://www.ncbi.nlm.nih.gov/geo/query/acc.cgi?acc=GSE81314 https://www.ncbi.nlm.nih.gov/geo/query/acc.cgi
