# Supplementary material for: Impact of 5HydroxyMethylCytosine (5hmC) on reverse/direct association of cell-cycle, apoptosis, and extracellular matrix pathways in gastrointestinal cancers
Source: BMC Genom Data. 2022 Jun 29;23:49. doi: 10.1186/s12863-022-01061-x (PMC9241275; doi:10.1186/s12863-022-01061-x)
Supplement: Supplementary file 1 — Additional file 1. [file 12863_2022_1061_MOESM1_ESM.docx]

**A Gastrointestinal Pan-Cancer Analysis of co-5hydroxymethylcytosine (co-5hmC) Network as an Epigenetic Mark Reveals a Promising Diagnostic Model for Distinguishing Cancer Patients and Normal Individuals**

The statistically significant modules detected in this study are GreenYellow, tan, and black. The list of genes in each module is reported in tables S1, S2, and S3. The module membership and gene significance of modules were reported in each table.

Table S1 GreenYellow

| geneModuleMembership | geneTraitSignificance | genelist |
| --- | --- | --- |
| 0.875716 | 0.359162 | BET1 |
| 0.965452 | 0.482019 | BMP2K |
| 0.831488 | 0.367969 | BTG1 |
| 0.851026 | 0.473823 | BUB3 |
| 0.841572 | 0.333947 | C12orf39 |
| 0.547122 | 0.29795 | C16orf82 |
| 0.900323 | 0.42027 | C18orf54 |
| 0.86755 | 0.351113 | C1orf170 |
| 0.939527 | 0.523145 | C21orf91 |
| 0.709475 | 0.43288 | C2orf27B |
| 0.925322 | 0.517513 | C4orf33 |
| 0.888572 | 0.392752 | C5orf51 |
| 0.953193 | 0.494914 | CD36 |
| 0.947946 | 0.489405 | CD55 |
| 0.72738 | 0.302161 | CDK3 |
| 0.593877 | 0.340325 | CDK5R2 |
| 0.718246 | 0.460908 | CDKN2C |
| 0.961939 | 0.453135 | CEP152 |
| 0.814311 | 0.380747 | CLDN19 |
| 0.781361 | 0.387544 | COX17 |
| 0.847759 | 0.391957 | CRISP3 |
| 0.955002 | 0.412476 | CYLD |
| 0.89519 | 0.449053 | DAZL |
| 0.894172 | 0.556002 | DBF4 |
| 0.643048 | 0.440018 | DMRTA2 |
| 0.801564 | 0.421093 | DNAJC9 |
| 0.906262 | 0.464612 | DR1 |
| 0.925874 | 0.476066 | EXTL2 |
| 0.896809 | 0.332555 | FAM111A |
| 0.90467 | 0.429146 | FBXO5 |
| 0.933654 | 0.444996 | FER |
| 0.950545 | 0.490089 | FGF7 |
| 0.625008 | 0.115741 | FLJ10038 |
| 0.671341 | 0.359078 | FOXF1 |
| 0.94915 | 0.524452 | G2E3 |
| 0.814436 | 0.338304 | GADD45A |
| 0.862314 | 0.536468 | GAPT |
| 0.911429 | 0.45211 | GMFB |
| 0.973806 | 0.511272 | GNAT3 |
| 0.89618 | 0.554388 | GUCY1B3 |
| 0.780833 | 0.299462 | HPCA |
| 0.843653 | 0.49537 | IFI44 |
| 0.828573 | 0.353201 | KBTBD8 |
| 0.773923 | 0.377987 | KIAA1161 |
| 0.96566 | 0.525034 | KIAA1468 |
| 0.927944 | 0.513411 | KIF20B |
| 0.853894 | 0.504003 | KIRREL2 |
| 0.741918 | 0.367859 | KLK15 |
| 0.836412 | 0.436387 | KLRF1 |
| 0.793848 | 0.526205 | KLRG1 |
| 0.748109 | 0.307316 | KRT86 |
| 0.496489 | 0.42273 | LOC100133461 |
| 0.869188 | 0.447128 | LOC100507217 |
| 0.645116 | 0.408866 | LOC145474 |
| 0.757007 | 0.461623 | LOC645166 |
| 0.971208 | 0.557358 | METTL15 |
| 0.747917 | 0.425514 | MRPS18C |
| 0.958896 | 0.506676 | MTHFD2L |
| 0.947972 | 0.413307 | MTPN |
| 0.907843 | 0.416214 | MYL12A |
| 0.963379 | 0.453449 | NAP1L1 |
| 0.701717 | 0.065494 | NBEAP1 |
| 0.958159 | 0.528922 | NHLRC2 |
| 0.928347 | 0.391795 | OGFRL1 |
| 0.968018 | 0.413303 | PDCD10 |
| 0.974294 | 0.539336 | PIK3C3 |
| 0.875122 | 0.346208 | POU1F1 |
| 0.972299 | 0.516191 | PRKACB |
| 0.866892 | 0.364881 | PTTG1 |
| 0.93603 | 0.349985 | RAB32 |
| 0.741105 | 0.407257 | RAET1G |
| 0.670523 | 0.401823 | RBP4 |
| 0.889325 | 0.434991 | RECQL |
| 0.957264 | 0.396916 | RIPK2 |
| 0.941796 | 0.474386 | RTN4 |
| 0.742097 | 0.352133 | RTP1 |
| 0.818896 | 0.519768 | SAMD11 |
| 0.801558 | 0.311158 | SAMD9 |
| 0.611389 | 0.294006 | SCGB2A2 |
| 0.832187 | 0.309399 | SETD5-AS1 |
| 0.811345 | 0.389479 | SGOL1-AS1 |
| 0.766292 | 0.326552 | SLC26A10 |
| 0.950113 | 0.502186 | SMC4 |
| 0.758154 | 0.576957 | SOD3 |
| 0.905518 | 0.375766 | TCTEX1D1 |
| 0.859387 | 0.506075 | TLR10 |
| 0.960745 | 0.49818 | TMEFF2 |
| 0.715887 | 0.405036 | TSSK3 |
| 0.86407 | 0.514265 | TYRP1 |
| 0.568075 | 0.440149 | UTP3 |
| 0.932905 | 0.507368 | VRK1 |
| 0.770776 | 0.490347 | WTIP |
| 0.765788 | 0.406726 | ZFAND5 |
| 0.773518 | 0.367957 | ZNF385D |

Table S2 tan

| geneModuleMembership | geneTraitSignificance | genelist |
| --- | --- | --- |
| 0.761244 | 0.356059 | AGT |
| 0.812879 | 0.285669 | AKR7A3 |
| 0.834492 | 0.328987 | ARHGEF19 |
| 0.81955 | 0.299181 | ASIC3 |
| 0.880445 | 0.505431 | ASPM |
| 0.558635 | 0.441327 | BMP7 |
| 0.763138 | 0.291959 | C1QL1 |
| 0.939572 | 0.412626 | C3orf58 |
| 0.676157 | 0.21549 | CALY |
| 0.836666 | 0.429805 | CCDC112 |
| 0.770312 | 0.177179 | CCDC71L |
| 0.940892 | 0.452887 | CCDC82 |
| 0.87457 | 0.540258 | CCNH |
| 0.890522 | 0.404377 | CEP44 |
| 0.570821 | 0.30518 | CLDND2 |
| 0.609734 | 0.266815 | CLPSL2 |
| 0.925329 | 0.331042 | CTNNB1 |
| 0.702946 | 0.365595 | DDX11L2 |
| 0.749036 | 0.2874 | DEFB129 |
| 0.537477 | 0.118024 | DIO3 |
| 0.667206 | 0.242264 | DLX4 |
| 0.886136 | 0.385392 | DUS4L |
| 0.844913 | 0.425594 | ENPP4 |
| 0.783744 | 0.577746 | EPM2AIP1 |
| 0.911164 | 0.365981 | ETAA1 |
| 0.904853 | 0.386375 | EXOC1 |
| 0.669309 | 0.316276 | FAM57B |
| 0.900456 | 0.412126 | FAM98B |
| 0.484458 | 0.350057 | FCRLB |
| 0.814799 | 0.383676 | FGF3 |
| 0.907919 | 0.47127 | FSBP |
| 0.673232 | 0.367315 | GATSL1 |
| 0.867344 | 0.385742 | GCA |
| 0.743007 | 0.487584 | GHRHR |
| 0.475986 | 0.370589 | GNRH2 |
| 0.907835 | 0.514038 | GOLT1B |
| 0.765041 | 0.357377 | GPR142 |
| 0.862899 | 0.373598 | HMGB1 |
| 0.721216 | 0.260469 | HMGN2 |
| 0.823129 | 0.431655 | KCNRG |
| 0.623581 | 0.531268 | KLK14 |
| 0.730978 | 0.301186 | KLRC4 |
| 0.784038 | 0.421419 | LHX3 |
| 0.786188 | 0.482175 | LOC100130275 |
| 0.668362 | 0.365595 | LOC100286793 |
| 0.713725 | 0.324533 | LOC100505658 |
| 0.852223 | 0.489249 | LOC100506134 |
| 0.637764 | 0.241547 | LRIT1 |
| 0.766278 | 0.372315 | MAL |
| 0.57513 | 0.353849 | MAP1LC3C |
| 0.94926 | 0.446274 | MAP3K7 |
| 0.854069 | 0.289091 | MAPK15 |
| 0.687136 | 0.236021 | MBNL1-AS1 |
| 0.9169 | 0.560721 | MFF |
| 0.692062 | 0.344043 | MIR155HG |
| 0.894135 | 0.407207 | MTBP |
| 0.537354 | 0.238373 | NAP1L5 |
| 0.77099 | 0.440727 | NDUFAF4P1 |
| 0.929703 | 0.334526 | NIPSNAP3B |
| 0.757285 | 0.274178 | NOTUM |
| 0.722653 | 0.314269 | ONECUT3 |
| 0.629013 | 0.313 | ORM1 |
| 0.88367 | 0.39497 | PARP8 |
| 0.972426 | 0.442118 | PHF20L1 |
| 0.972251 | 0.445891 | PHIP |
| 0.862157 | 0.417729 | PSMD6 |
| 0.93813 | 0.41326 | RAB11FIP2 |
| 0.960055 | 0.375691 | RAB14 |
| 0.925167 | 0.352401 | RAD21 |
| 0.85978 | 0.333089 | RAP2A |
| 0.975541 | 0.444897 | SATB1 |
| 0.629144 | 0.324636 | SFN |
| 0.793435 | 0.264841 | SLC12A2 |
| 0.961505 | 0.422111 | SLC25A36 |
| 0.864941 | 0.397782 | SLC38A2 |
| 0.892616 | 0.506934 | SLC39A10 |
| 0.710094 | 0.454478 | SPATA31C1 |
| 0.766162 | 0.404414 | SSX2IP |
| 0.851373 | 0.412008 | STX19 |
| 0.959412 | 0.424964 | SUCO |
| 0.852896 | 0.393593 | TEX15 |
| 0.728444 | 0.342516 | TIGD2 |
| 0.903594 | 0.290593 | TIPARP |
| 0.941688 | 0.469685 | TMEM170B |
| 0.676766 | 0.354654 | TMEM179 |
| 0.930371 | 0.374048 | TRMT1L |
| 0.640617 | 0.405552 | UGT2B28 |
| 0.960864 | 0.47154 | USP15 |
| 0.962103 | 0.484962 | USP47 |
| 0.783691 | 0.445547 | WASH2P |
| 0.935183 | 0.385402 | ZDHHC21 |

Table S3 black

| geneModuleMembership | geneTraitSignificance | genelist |
| --- | --- | --- |
| 0.803473 | 0.341418 | ABP1 |
| 0.909923 | 0.264871 | ACY3 |
| 0.926623 | 0.44729 | ADAM22 |
| 0.760235 | 0.35153 | ADAMDEC1 |
| 0.579857 | 0.387739 | ADAMTS2 |
| 0.886357 | 0.31131 | ADAMTSL5 |
| 0.645626 | 0.558861 | ADAT2 |
| 0.882341 | 0.489412 | ADCY1 |
| 0.95609 | 0.527558 | ADGB |
| 0.601058 | 0.316389 | ADO |
| 0.896512 | 0.31802 | AEBP1 |
| 0.712981 | 0.310028 | AGR3 |
| 0.710939 | 0.325133 | AGRP |
| 0.749782 | 0.461231 | AJUBA |
| 0.803205 | 0.414252 | ALG10 |
| 0.466795 | 0.357679 | ALG1L9P |
| 0.873667 | 0.222931 | ALG3 |
| 0.832816 | 0.384751 | ALKBH2 |
| 0.813761 | 0.220563 | ALKBH6 |
| 0.891406 | 0.343915 | ALKBH7 |
| 0.650217 | 0.226907 | ALPPL2 |
| 0.725259 | 0.278642 | AMBP |
| 0.867242 | 0.22003 | ANAPC11 |
| 0.887355 | 0.509749 | ANGPT1 |
| 0.786375 | 0.26016 | ANKLE1 |
| 0.843892 | 0.506916 | ANKRD18DP |
| 0.864363 | 0.241755 | ANKRD23 |
| 0.903676 | 0.503123 | ANKRD31 |
| 0.734242 | 0.286741 | ANKRD36 |
| 0.85922 | 0.434309 | ANKRD36BP2 |
| 0.867196 | 0.51865 | ANLN |
| 0.904396 | 0.301841 | ANO1 |
| 0.810429 | 0.399868 | ANXA1 |
| 0.661987 | 0.330766 | APH1A |
| 0.723888 | 0.22249 | APOC1P1 |
| 0.796617 | 0.329099 | APOL2 |
| 0.534744 | 0.368422 | ARF6 |
| 0.878507 | 0.336503 | ARFRP1 |
| 0.956671 | 0.479739 | ARHGAP15 |
| 0.888767 | 0.459626 | ARHGAP18 |
| 0.907525 | 0.372908 | ARHGAP33 |
| 0.915001 | 0.460868 | ARHGAP39 |
| 0.923875 | 0.369507 | ARHGEF10L |
| 0.92359 | 0.452923 | ARHGEF16 |
| 0.746699 | 0.273627 | ARID3C |
| 0.875777 | 0.259915 | ARMC6 |
| 0.712448 | 0.574766 | ARPC5 |
| 0.699131 | 0.173625 | ARTN |
| 0.90064 | 0.449618 | ASB6 |
| 0.862942 | 0.350908 | ASL |
| 0.74355 | 0.354797 | ASPDH |
| 0.89251 | 0.323478 | ASPG |
| 0.894652 | 0.327052 | ASS1 |
| 0.90387 | 0.432635 | ATOH8 |
| 0.827103 | 0.271204 | ATP2B2 |
| 0.910567 | 0.500766 | ATP2C1 |
| 0.612864 | 0.243012 | ATP5G1 |
| 0.795598 | 0.377971 | ATP6V0E2 |
| 0.925963 | 0.456722 | ATP8A1 |
| 0.792561 | 0.3214 | AVP |
| 0.889723 | 0.311075 | B3GAT1 |
| 0.731158 | 0.247336 | B3GNT9 |
| 0.601593 | 0.345145 | B4GALNT1 |
| 0.909431 | 0.550131 | BANK1 |
| 0.891817 | 0.30581 | BAP1 |
| 0.941004 | 0.512096 | BARD1 |
| 0.872205 | 0.500891 | BBS12 |
| 0.867139 | 0.258716 | BCL2L12 |

The coefficients for the predictive model were reported in Table S4. The G2E3, GNAT3, KIF20B, and KIRREL2 were not significant (Table S4).

Table S4

| (Intercept) |  |
| --- | --- |
| C21orf91 | 0.1993909 |
| C4orf33 | 2.8827925 |
| DBF4 | 9.3883328 |
| G2E3 | . |
| GAPT | 0.3466525 |
| GNAT3 | . |
| GUCY1B3 | 1.4137857 |
| KIAA1468 | -0.147651 |
| KIF20B | . |
| KIRREL2 | . |
| KLRG1 | 8.3757463 |
| METTL15 | . |
| MTHFD2L | -1.5265991 |
| NHLRC2 | . |
| PIK3C3 | . |
| PRKACB | . |
| SAMD11 | -1.1759777 |
| SMC4 | . |
| SOD3 | -9.8251817 |
| TLR10 | . |
| TYRP1 | . |
| VRK1 | -0.9218785 |

The preservation statistics were reported in the Table S5 and Table S6. The preservation statistics were computed for 25%, 50%, 75%, and GSE89570.

Table S5

|  | 50% | | | | 25% | | | |
| --- | --- | --- | --- | --- | --- | --- | --- | --- |
|  | s1.medianRank.pres | s1.medianRank.qual | s1.Zsummary.pres | s1.Zsummary.qual | s1.medianRank.pres | s1.medianRank.qual | s1.Zsummary.pres | s1.Zsummary.qual |
| black | 8 | 11 | 35 | 25 | 4 | 11 | 37 | 25 |
| blue | 13 | 12 | 42 | 23 | 8 | 12 | 38 | 23 |
| brown | 15 | 14 | 32 | 14 | 13 | 14 | 41 | 15 |
| cyan | 1 | 1 | 22 | 17 | 10 | 1 | 15 | 19 |
| gold | 14 | 15 | 32 | -1 | 14 | 15 | 37 | -0.58 |
| green | 11 | 2 | 39 | 48 | 1 | 2 | 47 | 48 |
| greenyellow | 7 | 9 | 15 | 15 | 7 | 9 | 12 | 13 |
| magenta | 12 | 6 | 17 | 20 | 6 | 6 | 16 | 18 |
| midnightblue | 3 | 4 | 16 | 17 | 2 | 4.5 | 15 | 16 |
| pink | 6 | 8 | 21 | 19 | 9 | 8 | 14 | 22 |
| purple | 3 | 3 | 21 | 18 | 5 | 3 | 16 | 21 |
| salmon | 4 | 7 | 17 | 14 | 11 | 7 | 10 | 16 |
| tan | 5 | 10 | 13 | 8.9 | 3 | 10 | 12 | 11 |
| turquoise | 10 | 5 | 57 | 56 | 10 | 4.5 | 55 | 58 |
| yellow | 9 | 13 | 38 | 14 | 15 | 13 | 32 | 14 |

Table S6

|  | 75% | | | | GSE89570 | | | |
| --- | --- | --- | --- | --- | --- | --- | --- | --- |
|  | s1.medianRank.pres | s1.medianRank.qual | s1.Zsummary.pres | s1.Zsummary.qual | s1.medianRank.pres | s1.medianRank.qual | s1.Zsummary.pres | s1.Zsummary.qual |
| black | 3 | 11 | 42 | 27 | 3 | 11 | 42 | 27 |
| blue | 9 | 12 | 45 | 24 | 13 | 12 | 45 | 24 |
| brown | 14 | 14 | 46 | 14 | 16 | 14 | 46 | 14 |
| cyan | 2 | 1 | 19 | 16 | 2 | 1 | 19 | 16 |
| gold | 15 | 15 | 38 | 1.7 | 15 | 15 | 38 | 1.7 |
| green | 11 | 2 | 47 | 42 | 12 | 2 | 47 | 42 |
| greenyellow | 6 | 9 | 16 | 15 | 6 | 12 | 18 | 15 |
| magenta | 12 | 6 | 18 | 19 | 12 | 6 | 18 | 20 |
| midnightblue | 4 | 4 | 20 | 14 | 4 | 5 | 20 | 14 |
| pink | 8 | 8 | 21 | 15 | 8 | 7 | 21 | 15 |
| purple | 3 | 3 | 23 | 20 | 3 | 4 | 23 | 14 |
| salmon | 7 | 7 | 16 | 17 | 7 | 7 | 17 | 17 |
| tan | 5 | 10 | 15 | 9.5 | 5 | 12 | 16 | 9.5 |
| turquoise | 10 | 5 | 59 | 60 | 12 | 5 | 59 | 60 |
| yellow | 13 | 13 | 38 | 14 | 13 | 13 | 38 | 14 |
